# Large Language Model–Based Analysis of Statin Therapy Discussions and Sentiment on Social Media: Cross-Sectional Observational Study

**DOI:** 10.2196/85057

**Published:** 2026-04-10

**Authors:** Siru Liu, Jialin Liu

**Affiliations:** 1 Department of Biomedical Informatics, Vanderbilt University Medical Center, 2525 West End Ave #1475, Nashville, TN, 610041, United States, 1 615-875-5216; 2 Department of Computer Science, Vanderbilt University, Nashville, TN, United States; 3 Department of Medical Informatics, West China Medical School, Chengdu, China; 4 Department of Otolaryngology-Head and Neck Surgery, West China Hospital, Sichuan University, Chengdu, China

**Keywords:** statin therapy, social media, large language model, sentiment analysis, Reddit, cardiovascular disease, patient perspectives, adherence, adverse effects, cohort study

## Abstract

**Background:**

Statin therapy, despite proven cardiovascular benefits, remains underused. Social media platforms may capture patient perspectives that are less visible in clinical encounters.

**Objective:**

This study aimed to characterize themes, sentiment, and decision-making factors related to statin therapy through large language model (LLM)–based analysis of Reddit discussions.

**Methods:**

This cross-sectional observational study analyzed English-language Reddit posts and comments mentioning statins from January 2022 to May 2025, identified via keyword-based Reddit application programming interface searches (≤1000 posts per keyword). A total of 5328 retrieved discussions (n=1661, 31.2% posts and n=3667, 68.8% keyword-containing comments) from public subreddits were included. Themes, sentiments (positive, neutral, or negative), guideline-informed clinical relevance, information-seeking behavior, adverse effect mentions, decision factors, and adherence-related content were extracted using an LLM-based pipeline.

**Results:**

Among 5328 discussions, prominent topics included adverse effects (n=1697, 31.9%), decision-making references related to laboratory results and physician advice (n=2767, 51.9% and n=2034, 38.2%, respectively), and alternative approaches (n=2485, 46.6%). Overall sentiment was neutral in 34% (n=1812) of discussions, negative in 30.9% (n=1646), and positive in 16.9% (n=900); the remainder were mixed or unclear. Statin-directed sentiment was neutral in 44.1% (n=2350) of discussions, negative in 25.2% (n=1343), and positive in 12.5% (n=666); the remainder did not express statin-directed sentiment. High clinical relevance was identified in 12.6% (n=672) of discussions. Adherence-related issues were mentioned in 29.8% (n=1587) of discussions. Among adverse effect mentions, muscle pain (n=129, 7.6%) and fatigue (n=110, 6.5%) were common.

**Conclusions:**

LLM-enabled analysis of Reddit discourse highlights substantial negative sentiment, adherence-related concerns, and adverse effect narratives surrounding statin therapy. These findings suggest opportunities for patient-centered communication and shared decision-making strategies that address symptom attribution, uncertainty, and information needs in digital information environments.

## Introduction

Statins are among the most widely prescribed medications globally, with more than 200 million individuals using these agents to reduce cardiovascular morbidity and mortality [1]. These 3-hydroxy-3-methylglutaryl coenzyme A reductase inhibitors remain the cornerstone of atherosclerotic cardiovascular disease (ASCVD) prevention, demonstrating well-established efficacy in both primary and secondary prevention settings [2,3]. Despite their proven benefits, real-world statin therapy is frequently limited by concerns regarding safety and tolerability. In routine care, statin-related events are commonly documented, with myalgia and myopathy representing the most frequent category, and these events often precipitate temporary discontinuation and subsequent rechallenge [4]. Consequently, long-term use remains suboptimal; a recent systematic review and meta-analysis found that only approximately 62% of patients achieve good adherence (≥80% use) over a median follow-up of 24 months [5], while population-based primary care data show frequent discontinuation with substantial restarting, consistent with an intermittent use pattern in practice [6].

Traditional clinical research methods, including randomized controlled trials and observational studies, may not fully capture the breadth of patient experiences and concerns in routine care [7]. Effective communication between patients and health care providers is essential for shared decision-making and treatment adherence; however, barriers such as limited consultation time, health literacy challenges, and patient reluctance to disclose adverse experiences may constrain insight into the real-world challenges of medication use [8,9]. Patients may hesitate to report symptoms or concerns to clinicians due to fear of judgment, desire to avoid conflict with prescribers, or uncertainty about whether their experiences warrant clinical attention [9]. Social media platforms, especially Reddit (Reddit, Inc), provide access to unfiltered patient narratives that may not be expressed during clinical encounters [10-13]. Reddit hosts a wide range of health-related communities and has more than 50 million daily active users [14]. Within these forums, individuals share personal medical experiences, seek peer advice, and discuss treatment decisions. These discussions may provide valuable insights into patient beliefs, motivations, and barriers that influence medication adherence and cardiovascular risk management [15-17].

Recent advances in large language models (LLMs) offer scalable methods to analyze such unstructured, high-volume textual data, enabling systematic characterization of patient perspectives at a granularity that would be infeasible using manual qualitative approaches alone [18]. In the context of cardiovascular prevention, applying LLMs to social media discourse may provide novel insights into how patients interpret statin-related information, evaluate perceived risks and benefits, and make adherence decisions outside the clinical setting. Accordingly, this study aimed to systematically analyze Reddit discussions related to statin use using an LLM to characterize patient-reported experiences, identify recurrent concerns and misconceptions, and explore factors influencing decision-making and adherence. By integrating patient-generated narratives with computational text analysis, this work seeks to complement traditional evidence sources and inform more patient-centered approaches to cardiovascular risk management.

## Methods

### Study Design and Data Source

This cross-sectional study analyzed publicly available Reddit discussions about statin therapy posted between January 1, 2022, and May 1, 2025. Reddit was selected because it hosts large, topic-specific health communities in which users openly share medication experiences, treatment decisions, and interactions with health care providers in naturalistic settings. Data were accessed via the official Reddit application programming interface (API) using Python Reddit API Wrapper, limited to public subreddits, and no user contact or reidentification attempts were made.

### Data Collection

#### Search Strategy

We retrieved content using “statin,” generic statin names (eg, atorvastatin, rosuvastatin, simvastatin, pravastatin, lovastatin, fluvastatin, and pitavastatin), and US brand names (eg, Lipitor, Crestor, Zocor, Pravachol, Livalo, Lescol, and Mevacor); full queries are provided in Multimedia Appendix 1.

#### API Procedure

Using Python Reddit API Wrapper (version 7.7.1) in Python (version 3.9; Python Software Foundation; May 15-May 20, 2025), we queried Reddit’s search end point (sorted by “new” where available). Due to indexing and API constraints, each keyword returned up to 1000 submissions. For each submission, we extracted metadata (submission ID, created_utc, subreddit, title, selftext, author, score, comment count, and permalink) and downloaded its comment tree (comment ID, parent ID, created_utc, author, body, and score). We retained submissions within the study window; pagination, time stamps, and rate-limit handling are described in Multimedia Appendix 1.

#### Eligibility

We included English-language posts and comments from public subreddits that matched 1 or more keywords (case-insensitive) within the study window and excluded removed and deleted placeholders, duplicates, non-English text (langdetect v1.0.9), and promotional and spam content (Multimedia Appendix 1).

#### Unit of Analysis

The unit of analysis was a Reddit discussion thread (eligible submission plus associated comments), enabling contextual interpretation of patient narratives and peer responses and supporting downstream LLM-based thematic extraction [19].

### Data Cleaning and Preprocessing

We removed URLs and normalized whitespace while preserving the original wording (no stemming or lemmatization). Privacy protection included removing @mentions and replacing detected email addresses and phone numbers with placeholders (eg, “[EMAIL],” “[PHONE]”); subreddit names were retained as public metadata, with reidentification risk minimized via aggregate reporting and avoidance of traceable quotations [20]. English language posts were identified using fastText (lid.176.bin) [21] (retain ≥0.80, adjudicate 0.60-0.80 by 2 investigators, and exclude <0.60). For analysis, comments were retained if they contained 1 or more keywords; misspellings and variants were captured via regex-based partial matching with guardrails. Exact duplicates were removed via SHA-256 hashing, and near duplicates were excluded using MinHash with Jaccard similarity ≥0.85 [22]. All steps were deterministic and version controlled; a PRISMA (Preferred Reporting Items for Systematic Reviews and Meta-Analyses)–style flow diagram is provided in Multimedia Appendix 1.

### LLM-Based Content Extraction

Structured extraction used GPT-4.1 [23] via the OpenAI API (version 1.52.0) between May and June 2025 with standardized parameters (temperature=0.1, top_p=1.0, and max_tokens=4096). A single prompt template and predefined JSON schema were applied to all discussions. Two investigators iteratively developed the schema and decision rules through pilot testing on 50 purposively sampled discussions and consensus refinement of edge cases, consistent with clinical reasoning standards [18,24]. For 11 analytic domains (Table 1), outputs included structured variables, brief summaries, and verbatim evidence quotes supporting key classifications. Dietary changes and exercise were coded as alternative approaches only when explicitly discussed as substitutes for statin therapy (eg, attempting to avoid, delay, or discontinue statins); when described as adjuncts alongside statins, they were not classified as alternatives. Outputs failing JSON validation or missing mandatory evidence were automatically requeried up to 3 times; only schema-valid outputs were retained (Multimedia Appendix 2).

**Table 1.** GPT-4.1 content analysis framework with examples.

Domain	Description	Example extractions
Primary themes	1 to 3 main discussion topics	Concerns about statin adverse effects, cholesterol management through lifestyle changes, and physician-patient communication
Statin medications	Specific statin names mentioned	Generic: atorvastatin and rosuvastatin Brand: Lipitor and Crestor
Experience type	Postclassification	Personal experience, information request, and medical advice
Sentiment analysis	Multitarget emotional assessment	Neutral, negative, or positive Overall, toward statins and toward physicians
Clinical relevance	Actionable insight assessment based on AHA^a^ and ACC^b^ guidelines	High: ASCVD^c^ history (MI^d^, stroke, and PAD^e^), LDL-C^f^ ≥190 mg/dL, diabetes (in individuals aged 40‐75 years), and 10-year ASCVD risk ≥7.5% Medium: family history of premature ASCVD, chronic kidney disease, inflammatory conditions, CAC^g^ score ≥100, and smoking Low: general cardiovascular health discussions
Adverse effects	Adverse reactions mentioned	Muscle pain, fatigue, brain fog, dizziness, nausea, and memory issues
Decision factors	Treatment choice influences	Laboratory results, physician recommendation, and adverse effects
Information seeking	Behavioral patterns	Asking for advice, sharing experience, and seeking alternatives
Adherence issues	Medication compliance	Mentions discontinuation, compliance issues, and dose changes
Alternative treatments	Nonstatin options	Diet changes, exercise, ezetimibe, and coenzyme Q10
Emotional indicators	Expressed emotions	Frustration, confusion, hope, and anxiety

a AHA: American Heart Association.

b ACC: American College of Cardiology.

c ASCVD: atherosclerotic cardiovascular disease.

d MI: myocardial infarction.

e PAD: peripheral artery disease.

f LDL-C: low-density lipoprotein cholesterol.

g CAC: coronary artery calcium.

All content was analyzed as user-generated narratives. References to clinician recommendations within discussions (eg, “my doctor recommended starting a statin”) were coded as patient-reported physician communication rather than independently verified clinical input. Posts containing general medical guidance without a described patient-clinician interaction were not coded under the “decision factors” physician recommendation category. Clinician perspectives were not independently ascertained; physician-related content was captured only through users’ own accounts of clinical interactions.

### Clinical Relevance Tiering

A deterministic rule-based algorithm aligned with American Heart Association, American College of Cardiology, and US Preventive Services Task Force indicators [25,26] assigned high, medium, or low clinical relevance (Multimedia Appendix 2). High relevance required explicit mention of ASCVD, low-density lipoprotein cholesterol ≥190 mg/dL, diabetes (in individuals aged 40‐75 years), or elevated 10-year ASCVD risk, and medium relevance reflected risk-enhancing factors (eg, family history of premature ASCVD, chronic kidney disease, inflammatory conditions, and coronary artery calcium ≥100 Agatston units) [25,26]; otherwise, discussions were low relevance. Given self-reported data, absence of criteria was coded as “unreported,” and tier assignment defaulted downward unless explicit evidence supported a higher tier.

### Detection of LLM-Related Content

We searched for mentions of LLM tools (eg, ChatGPT [OpenAI], Claude [Anthropic], and Google Gemini) and categorized them as (1) seeking medical advice, (2) seeking general health information, (3) sharing LLM-generated content, or (4) incidental mentions. Categories 1 to 3 were flagged for sensitivity analyses (Multimedia Appendix 2).

### Validation

LLM outputs were validated via expert review (Multimedia Appendix 3). A stratified random sample of 50 discussions (0.9% of 5328) spanning key domains was independently assessed by 2 reviewers (a clinical informaticist and a physician) using a 5-point accuracy scale (1=very poor to 5=excellent). Interrater agreement used Cohen κ. Reviewers also documented recurrent errors. Verbatim evidence requirements enabled direct verification of extracted labels.

### Statistical Analysis

Multivariable logistic regression identified factors associated with negative sentiment toward statins. Cluster-robust SEs were estimated at the subreddit level. Covariates were prespecified (clinical relevance tier, adverse effect mentions, adherence-related content, information-seeking behavior, specific statins, and subreddit category). Absence of a mention was coded as “not reported.” Diagnostics included variance inflation factors and influential observation checks. Results are reported as odds ratios with 95% CIs; 2-sided *P*<.05 indicated significance.

### Ethical Considerations

This study analyzed publicly accessible Reddit posts and comments as secondary data and involved no direct contact, interaction, intervention, or attempt to reidentify users. Informed consent was not obtained because the study used publicly available online content, the investigators did not interact with users, and obtaining consent from all posters was not feasible. No compensation was provided because no participants were recruited or contacted. The study was exempt from institutional review board approval and aligned with the Association of Internet Researchers’ Internet Research: Ethical Guidelines 3.0 [27].

## Results

### Dataset Characteristics and Distribution

After systematic filtering, the final dataset comprised 5328 discussions contributed by 4832 unique users across multiple Reddit communities. The most frequently represented forum was r/Cholesterol (n=2722, 51.1%). Nearly half of the discussions (n=2552, 47.9%) referenced a specific statin. The most commonly mentioned agents were rosuvastatin (n=1276, 23.9%) and atorvastatin (n=1013, 19%). Dataset characteristics and statin mentions are summarized in Table 2.

**Table 2.** Characteristics of Reddit discussions and statin mentions (N=5328).

Characteristic and category		Mentions, n (%)	
Document type			
Submissions (posts)		1661 (31.2)	
Comments		3667 (68.8)	
Source community			
r/Cholesterol		2722 (51.1)	
r/PeterAttia		576 (10.8)	
r/AskDocs		300 (5.6)	
r/keto		92 (1.7)	
r/HeartAttack		87 (1.6)	
r/diabetes_t2		82 (1.5)	
r/stroke		46 (0.9)	
Other subreddits		1423 (26.7)	
Primary discussion type			
Personal experience sharing		3210 (60.2)	
Information request		882 (16.6)	
Medical advice provision		734 (13.8)	
General discussion		502 (9.4)	
Mention of a specific statin			
Yes		2552 (47.9)	
No		2776 (52.1)	
Most frequent statins^a^			
Rosuvastatin (Crestor)		1276 (23.9)	
Atorvastatin (Lipitor)		1013 (19)	
Pravastatin (Pravachol)		279 (5.2)	
Simvastatin (Zocor)		211 (4)	

a Generic and brand name mentions were combined.

### Thematic Analysis and Primary Discussion Topics

**Figure 1.**
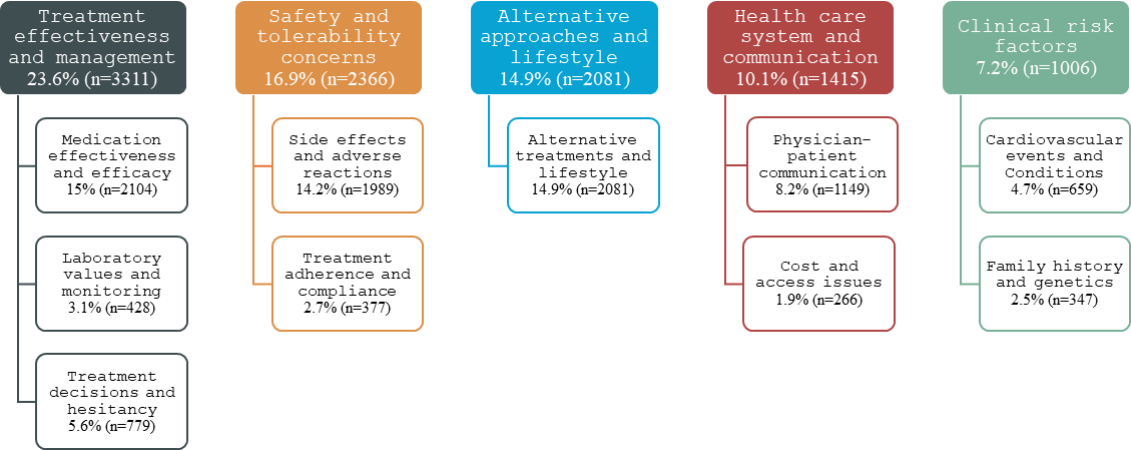
Quantitative distribution of themes in Reddit discussions related to statins (January 2022 to May 2025). The hierarchical tree map illustrates the primary discussion topics identified by the large language model pipeline across 5328 discussions, organized into 5 overarching thematic groups and 10 topic categories. Each discussion could receive up to 3 thematic codes; percentages represent the proportion of total thematic mentions (N=14,007). The 10 most frequent topic categories are shown; remaining thematic mentions (n=3828, 27.3%) comprised less frequent topics and are not displayed.

Thematic analysis showed that discussions most commonly focused on treatment effectiveness (3311/14,007, 23.6% of thematic mentions), followed by safety and tolerability concerns (n=2366, 16.9%) and alternative lifestyle interventions (n=2081, 14.9%; Figure 1). Privacy-preserving, paraphrased exemplar posts (1‐2 per major theme) and the corresponding LLM theme classifications are provided in Multimedia Appendix 4.

### Sentiment Analysis

Overall sentiment was neutral in 34% of discussions, negative in 30.9%, and positive in 16.9%, with the remainder classified as mixed or unclear. Sentiment distributions varied significantly by community type (*χ_8_*²=335.7; *P*<.001). The proportion of discussions with negative sentiment was substantially higher in medical advice–seeking forums (eg, r/AskDocs: 77.3%) than in lifestyle-oriented forums (eg, r/Biohackers: 9.1%).

### Clinical Relevance Assessment

Among 5328 discussions, using guideline-informed criteria, 12.6% (n=672) of discussions were classified as high clinical relevance, 22.4% (n=1193) as medium relevance, and 65% (n=3463) as low relevance. High-relevance content was most frequent in r/stroke (40/46, 87.0%), r/HeartAttack (41/87, 47.1%), r/diabetes_t2 (29/82, 35.4%), and r/AskDocs (82/300, 27.3%). Clinical information elements were reported as follows: laboratory values (n=2048, 38.4%), cardiovascular events (n=362, 6.8%), family history (n=908, 17%), and lifestyle factors (n=2616, 49.1%).

### Adverse Effect Reports and Safety Concerns

Among 5328 discussions, adverse effects were reported in 31.9% (n=1697; 95% CI 30.7%‐33.2%) of discussions. Among discussions that referenced adverse effects, the most frequent included muscle pain (n=129, 7.6%) and fatigue (n=110, 6.5%), followed by cognitive symptoms such as “brain fog” (n=61, 3.6%). A detailed breakdown of adverse effects, including neuropsychiatric symptoms, is provided in Multimedia Appendix 5. In 0.3% (n=17) of discussions, users explicitly stated that no adverse effects occurred.

### Decision-Making Factors and Treatment Influences

Of 5328 discussions, the most frequently coded factors associated with statin-related decisions were laboratory results (n=2767, 51.9%), physician recommendations (n=2034, 38.2%), adverse effects (n=1593, 29.9%), and family history of cardiovascular disease (n=822, 15.4%). Additional factors included lifestyle modifications (n=1092, 20.5%), online research (n=573, 10.8%), cost (n=316, 5.9%), and insurance coverage (n=72, 1.4%). Genetic predisposition and clinical guideline recommendations were each coded in 0.5% (n=26 for both) of discussions.

### Alternative Treatment Discussions

Among 5328 discussions, alternative treatments were coded in 46.6% (n=2485) of discussions. The most frequent nonpharmacologic alternatives were dietary changes (n=449, 8.4%) and exercise (n=406, 7.6%). Pharmacologic alternatives included ezetimibe (n=168, 3.2%), Repatha (n=84, 1.6%), and Zetia (n=70, 1.3%). Supplements included fish oil (n=60, 1.1%), coenzyme Q10 (n=57, 1.1%), and red yeast rice (n=53, 1%). Weight loss was coded in 1.4% (n=73) of discussions.

### Information-Seeking Behaviors and Community Engagement

Among 5328 discussions, experience sharing was coded in 78.2% (n=4167) of discussions and advice seeking was coded in 46.1% (n=2456). Discussions focused on alternative treatments occurred in 7.2% (n=386) of cases, while 5.3% (n=284) included questioning medication necessity. Advice seeking was most frequent in r/AskDocs (n=292, 97.3%).

### Emotional Indicators and Adherence Issues

Of the 5328 discussions, emotional expressions were identified in 85.2% (n=4537) of discussions. The most frequently coded emotions were frustration (n=1705, 32%) and confusion (n=911, 17.1%). Medication adherence issues were present in 29.8% (n=1587) of the discussions, including discontinuation (n=522, 9.8%) and dose modification (n=437, 8.2%). Multiple codes per discussion were permitted; therefore, percentages may sum to more than 100%.

### Mentions of LLM Tools in Reddit Discussions

Explicit mentions of LLM tools occurred in 23/5,328 (0.4%) discussions, totaling 38 mentions. Of the 38 mentions, ChatGPT accounted for 32 (84.2%), followed by other GPT variants (n=3, 7.9%) and Claude, Bard/Gemini, or DeepSeek (n=3, 7.9% combined). Mentions were coded as statin-related medical decision-making (n=2, 5.3%), information seeking (n=6, 15.8%), and general references (n=30, 78.9%). Discussions containing LLM mentions were most commonly observed in r/PeterAttia (6/23, 26.1%) and r/Cholesterol (5/23, 21.7%). Mentions increased from 6 in 2023 to 32 in January 2025 to May 2025.

### Expert Validation Results

Expert validation of a stratified sample of 50 discussions showed a mean validation score of 4.67 (SD 0.74). Interrater agreement was substantial (Cohen κ=0.85; 95% CI 0.78-0.92). Expert feedback noted recurring coding challenges in distinguishing genetic risk factors (eg, lipoprotein[a]) from family history, as well as in identifying cardiovascular event–related content (eg, coronary artery calcium scores).

### Statistical Associations

Sentiment distributions differed across communities (*χ*²=335.7; *P*<.001). Negative sentiment was more frequent in medical advice–seeking communities than in lifestyle-focused communities (eg, r/AskDocs: 77.3% vs r/Biohackers: 9.1%; *P*<.001). Adverse effect reporting also differed by community type (*χ_2_*²=124.6; *P*<.001). Adverse effect mentions and adherence issues were associated with negative sentiment (*P*<.001 for both). In adjusted multivariable analysis, factors associated with negative sentiment included adverse effect mentions (adjusted odds ratio [aOR] 3.42, 95% CI 2.89‐4.05; *P*<.001), posts in medical advice communities (aOR 2.76, 95% CI 2.31‐3.30; *P*<.001), adherence issues (aOR 2.18, 95% CI 1.84‐2.58; *P*<.001), and high clinical relevance content (aOR 1.47, 95% CI 1.18‐1.84; *P*=.001; Table 3).

**Table 3.** Multivariable logistic regression of factors associated with negative sentiment.

Variable	Adjusted odds ratio (95% CI)		*P* value
Adverse effect mentions	3.42 (2.89‐4.05)		<.001
Posts in medical advice communities	2.76 (2.31‐3.30)		<.001
Adherence issues	2.18 (1.84‐2.58)		<.001
High clinical relevance content	1.47 (1.18‐1.84)		.001

## Discussion

### Main Findings

In this large-scale analysis of statin-related discussions on Reddit, patient discourse was shaped primarily by perceived adverse effects, uncertainty about benefits, and peer validation, with relatively limited reference to formal cardiovascular risk stratification. Although statins are strongly endorsed by clinical guidelines for ASCVD prevention [2,26], real-world patient narratives were frequently framed around experiential and emotional factors, including fear of long-term harm, symptom attribution, and ambivalence toward medical authority. This divergence between guideline-based evidence and patient-centered concerns helps explain persistently suboptimal statin adherence despite decades of robust trial data [28].

Notably, fewer than 1 in 8 discussions met criteria for high clinical relevance based on American Heart Association, American College of Cardiology, and US Preventive Services Task Force risk thresholds [2,26]. Instead, most conversations focused on nonspecific symptoms, laboratory fluctuations, or lifestyle considerations, suggesting that engagement with statin therapy often occurs outside a formal risk-benefit calculus. Therefore, effective statin counseling may require attention to patients’ beliefs and concerns, beyond clinical risk factors alone.

### The Adverse Effect Paradox: Perception vs Trial Evidence

Adverse effect concerns were prominent in our dataset (n=1697, 31.9% of discussions), which is not directly comparable to trial-based incidence estimates but may reflect a perception-evidence gap that shapes beliefs and adherence decisions. Evidence from blinded randomized trials and individual-participant meta-analyses suggests that the excess risk of muscle symptoms attributable to statins is small and that most muscle symptom reports under blinded conditions are not attributable to statin therapy [29]. This divergence is consistent with nocebo-related expectation and attribution mechanisms within the broader information environment. In the Anglo-Scandinavian Cardiac Outcomes Trial–Lipid-Lowering Arm, muscle-related adverse events were reported more frequently during the unblinded extension than during blinded treatment despite comparable exposure, supporting expectation-driven symptom attribution [30].

Cognitive concerns (eg, “brain fog”) appeared in 3.6% (n=61) of discussions, although randomized trials have not demonstrated statin-attributable cognitive impairment [31]. Regulatory agencies have noted rare, generally reversible postmarketing reports, and the prominence of such concerns online may reflect expectation-driven symptom attribution [32]. Mechanistically, true statin myopathy occurs in a subset of patients, including those with *SLCO1B1* variants or drug-drug interactions, but the high salience of adverse effects in online communities likely reflects both heterogeneity in susceptibility and selection effects (symptomatic users preferentially posting) [33,34]. Clinicians should anticipate that patients initiate statins within an information ecosystem that can magnify harm narratives. Proactive counseling, clear differentiation of evidence-based risks, and structured symptom assessment (eg, Statin-Associated Muscle Symptom Clinical Index) may reduce nocebo-driven discontinuation [35,36].

### Emotional Burden as a Hidden Driver of Nonadherence

Beyond physical symptoms, statin discussions carried substantial emotional content (n=4537, 85.2%), with frustration (n=1705, 32%) and confusion (n=911, 17.1%) predominating. These patterns suggest that statin decisions may be shaped by emotional responses (eg, frustration and anxiety) in addition to objective risk appraisal. Negative emotion is consistently associated with poorer adherence across chronic disease contexts. A meta-analysis by DiMatteo et al [37] found markedly higher nonadherence among patients with depression. Similarly, medication-specific emotional distress has been associated with treatment discontinuation, even after accounting for clinical depression [38,39]. In our dataset, adherence problems (n=1587, 29.8%) frequently co-occurred with adverse effect narratives and negative sentiment, suggesting a pathway in which perceived harms generate distress that undermines persistence. These emotional dimensions may be underelicited in routine care, where time-constrained visits often prioritize biomarker review and dose adjustment. Incorporating a brief assessment of medication-related distress into cardiovascular prevention workflows, paired with motivational interviewing, could identify patients at risk for emotionally driven discontinuation earlier in the treatment course.

### Community as an Information Filter: The Ecology of Online Health Discourse

Sentiment and content varied substantially across Reddit communities. Negative sentiment was more prevalent in medical advice–seeking subreddits (eg, r/AskDocs: 77.3%) than in lifestyle-focused subreddits (eg, r/Biohackers: 9.1%). Condition-specific forums (eg, r/stroke and r/HeartAttack) contained the highest proportion of clinically relevant content, while dietary communities (eg, r/keto) more often reflected cholesterol skepticism that diverged from guideline framing [2].

These patterns are consistent with community selection as an “information filter,” in which users preferentially encounter narratives aligned with prevailing community norms and their own concerns; similar dynamics have been described in other online health communities [40,41]. Such community-specific patterns may reinforce users’ existing perspectives. For example, dietary forums showed more skepticism toward statins, while condition-specific communities (eg, r/HeartAttack) contained more secondary prevention content. Therefore, community-specific outreach may be more effective than generic education. Strategies could include engaging trusted voices within skeptical forums, tailoring evidence presentation to community values, and directly addressing prevalent misconceptions (eg, “cholesterol myth” narratives), thereby complementing traditional patient counseling.

### The Emerging Role of Artificial Intelligence in Patient Decision-Making

Explicit mentions of LLMs were uncommon (n=23, 0.4%) but increased from 6 in 2023 to 32 in early 2025, suggesting growing uptake. Users described using tools such as ChatGPT to interpret laboratory results, weigh treatment options, and prepare questions for clinical encounters. To the best of our knowledge, few studies have quantified artificial intelligence (AI) tool use within real-world cardiovascular treatment discussions. Prior research has evaluated LLM performance on medical queries [42,43], but implications for patient expectations and decision-making remain incompletely characterized. These findings raise questions about how AI-mediated information may shape patient perspectives before clinical visits, warranting further investigation and practical guidance for clinicians.

### Limitations

This study has several limitations. First, Reddit API retrieval imposes sampling constraints. Search results are not uniformly sampled. Each keyword query is capped (≤1000 submissions), and ranking algorithms can overrepresent newer posts. This may yield a recency- and visibility-biased corpus. Accordingly, theme and sentiment frequencies should be interpreted as visibility-weighted patterns within the retrieved dataset rather than population-level prevalence for all statin-related content on Reddit; this may inflate high-salience topics (eg, adverse effects, discontinuation, and emotionally charged narratives) and complicate temporal comparisons. Second, Reddit’s pseudonymous structure prevents verification of demographics, diagnoses, lipid values, comorbidities, and outcomes. Users are unlikely to be representative of the broader statin-using population, limiting generalizability—particularly to older adults and those with lower digital access or health literacy. Third, self-reported social media content is subject to recall bias and selective posting, potentially overrepresenting unusual or negative experiences. Fourth, although we used expert-guided prompt development and validation, LLM-based extraction may misclassify nuanced language (eg, sarcasm or irony) or clinical attribution, which could affect domain-specific estimates. Evidence-quote requirements and human validation mitigate but do not eliminate this risk. Fifth, findings are limited to 1 platform and English-language content; discourse may differ across platforms and languages. Finally, this cross-sectional design cannot establish causality, assess within-person changes, or link discourse to clinical outcomes. Because nondisclosure in social media reflects “unreported” rather than missing at random, our conservative handling of unreported variables may underestimate clinical relevance and attenuate associations. We mitigate these limitations through transparent reporting, stratified expert validation, and emphasis on associations rather than causal claims. Future work should consider time-stratified sampling, multiplatform triangulation, and linkage to external clinical data (eg, registries, claims, or electronic health record–linked cohorts) where feasible.

### Conclusions

This study highlights a substantial misalignment between guideline-based statin risk stratification and the lived experiences expressed by patients online. Across 5328 Reddit discussions, discourse centered on perceived adverse effects (n=1697, 31.9%), emotional distress (n=4537, 85.2%), and mentions of “natural” alternatives (n=2485, 46.6%), patterns that may help explain persistent challenges in long-term adherence. These findings suggest that effective cardiovascular prevention must go beyond information provision to address nocebo effects and treatment-related frustration that shape patients’ responses to therapy. Methodologically, our validated LLM-enabled pipeline captures nuanced dimensions of treatment experience (eg, ambivalence and peer influence) that are poorly measured by conventional surveillance approaches. As patients increasingly navigate AI-mediated information environments, evidence-informed engagement with digital spaces may support shared decision-making and improve long-term outcomes.

## Supplementary material

10.2196/85057 Multimedia Appendix 1 Data cleaning and filtering.

10.2196/85057 Multimedia Appendix 2 Large language model–based extraction protocol and prompt.

10.2196/85057 Multimedia Appendix 3 Expert validation protocol.

10.2196/85057 Multimedia Appendix 4 Example posts with large language model–derived theme and sentiment classifications.

10.2196/85057 Multimedia Appendix 5 Frequency of specific adverse effects mentioned in Reddit discussions on statin therapy.

## Abbreviations

AI: artificial intelligence
aOR: adjusted odds ratio
API: application programming interface
ASCVD: atherosclerotic cardiovascular disease
LLM: large language model
PRISMA: Preferred Reporting Items for Systematic Reviews and Meta-Analyses
