# Supplementary material for: Large Language Model–Based Analysis of Statin Therapy Discussions and Sentiment on Social Media: Cross-Sectional Observational Study
Source: J Med Internet Res. 2026 Apr 10;28:e85057. doi: 10.2196/85057 (PMC13068305; doi:10.2196/85057)
Supplement: Multimedia Appendix 1 [file jmir-v28-e85057-s001.docx]

**Multimedia Appendix 1**

**Data Cleaning and Filtering**

We applied systematic data cleaning procedures to improve data quality: (1) Text cleaning to remove URLs, markdown formatting, non-ASCII characters, and excessive whitespace; (2) Deduplication to remove identical posts and comments; (3) Bot filtering to exclude automated accounts (AutoModerator and accounts containing bot); (4) Personal experience filtering to include only posts containing first-person language (I or my), ensuring focus on patient perspectives rather than general medical information; (5) Content enrichment using Reddit API to obtain engagement metrics (scores, comment counts) for submissions and comments.
