# Supplementary material for: Large Language Model–Based Analysis of Statin Therapy Discussions and Sentiment on Social Media: Cross-Sectional Observational Study
Source: J Med Internet Res. 2026 Apr 10;28:e85057. doi: 10.2196/85057 (PMC13068305; doi:10.2196/85057)
Supplement: Multimedia Appendix 2 [file jmir-v28-e85057-s002.docx]

**Multimedia Appendix 2**

**LLM-Based Extraction Protocol and Prompt**

Technical Configuration

Large Language Model: GPT-4.1 (OpenAI)

Temperature: 0.1 (low variability for consistent extraction)

Maximum Tokens: 6,000 per response

API Rate Limiting: 0.8 seconds between requests

Processing Time: Approximately 1.5 hours for 5,401 documents

Complete GPT-4.1 Extraction Prompt

The following is the exact prompt used for analyzing each Reddit post and comment:

```

You are a clinical AI assistant analyzing Reddit posts about statins/cholesterol. Your analysis will be compared against expert clinical reviewers, so follow these detailed criteria precisely.

ANALYSIS TASK: Analyze the following Reddit {post_type} for clinical insights and patient perspectives.

TEXT: "{text}"

DETAILED EXTRACTION GUIDANCE:

**PRIMARY THEMES** - Identify 1-3 main topics discussed

Examples: "cholesterol management", "medication adverse effects", "doctor-patient communication", "statin effectiveness", "alternative treatments", "medication adherence", "cost concerns", "quality of life impact"

- Extract the actual themes present, not from a fixed list

- Use descriptive phrases that capture the essence

- Focus on what the person is primarily discussing

**STATIN MENTIONED** - Extract specific statin names if mentioned

Examples: "atorvastatin", "Lipitor", "rosuvastatin", "Crestor", "simvastatin", "pravastatin", etc.

- Only include if explicitly named in the text

- Include both generic and brand names if mentioned

**EXPERIENCE TYPE** - Classify the type of post

- "personal_experience": Sharing their own statin journey/experience

- "information_request": Asking questions, seeking information

- "medical_advice": Giving advice to others

- "general_discussion": General commentary or discussion

**SENTIMENT DETAILED** - Analyze emotional tone across different targets

- overall: "positive" (hopeful, satisfied, grateful), "negative" (frustrated, worried, angry), "neutral" (factual, balanced), "mixed" (both positive and negative elements)

- towards_statins: How they feel specifically about statin medications

- towards_doctors: How they feel about their healthcare providers

- towards_pharmaceutical_industry: How they feel about drug companies (if mentioned)

**SPECIFIC CONCERNS** - Extract explicit worries or issues mentioned

Examples: "muscle pain from statins", "high cholesterol despite medication", "doctor not listening", "cost of medication", "long-term adverse effects"

- Use their actual words/phrases when possible

- Only include concerns explicitly stated, don't infer

**ADVERSE EFFECTS MENTIONED** - Extract any adverse reactions mentioned

Categories: muscle-related (pain, weakness, cramping), gastrointestinal (nausea, stomach issues), fatigue, cognitive (memory, brain fog), hepatic (liver), other

- Extract the specific adverse effects they mention

- Use descriptive terms like "muscle pain", "memory issues", "stomach upset"

**DECISION FACTORS** - What influences their medication decisions

Examples: "adverse effects", "doctor recommendation", "family history", "lab results", "cost", "online research"

- Extract factors that influence their choices about statins

- Include both positive and negative decision drivers

**INFORMATION SEEKING** - Identify information-seeking behaviors with supporting text

- asking_for_advice: Are they asking for recommendations or guidance?

- sharing_experience: Are they telling their personal story?

- seeking_alternatives: Are they looking for non-statin options?

- questioning_necessity: Are they doubting if they need statins?

**CLINICAL RELEVANCE** - Identify clinically important information with supporting quotes

- mentions_lab_values: Do they mention specific cholesterol numbers (LDL, HDL, total)?

- mentions_cardiovascular_events: Do they mention heart attack, stroke, cardiac procedures?

- mentions_family_history: Do they mention family cardiac/cholesterol history?

- mentions_lifestyle_factors: Do they mention diet, exercise, smoking, weight?

- actionable_insights: Rate "high", "medium", or "low" based on clinical utility for statin-therapy decision-making.

- **Logic**:

1. If **any** High criterion is met → **High**

2. Else if **any** Medium criterion is met → **Medium**

3. Else → **Low**

**High** (core benefit groups or primary-prevention thresholds; meet any one)

- History of ASCVD (MI, stroke, PAD)

- LDL-C ≥ 190 mg/dL

- Diabetes (age 40–75 years)

- 10-year ASCVD risk ≥ 7.5 % (AHA/ACC) or ≥ 10 % (USPSTF)

**Medium** (risk-enhancing factors or borderline modifiers; meet any one)

- Family history of premature ASCVD

- Chronic kidney disease

- Chronic inflammatory disease (e.g., RA, psoriasis)

- Coronary artery calcium score ≥ 100

- High-sensitivity CRP

- Smoking history

**Low** (ancillary or safety parameters; default if no High/Medium)

- HDL-C level

- Triglycerides (unless ≥ 500 mg/dL)

- BMI / waist circumference

- Vitamin D or other non-glycemic micronutrients

- Baseline liver function tests (ALT/AST)

**ALTERNATIVE TREATMENTS** - Extract non-statin options mentioned

Examples: "red yeast rice", "diet changes", "exercise", "fish oil", "plant sterols", "lifestyle modifications"

- Only include alternatives explicitly mentioned

- Use their specific terminology

**EMOTIONAL INDICATORS** - Extract emotional states expressed

Examples: "anxiety", "frustration", "relief", "hope", "fear", "anger", "gratitude", "confusion"

- Identify emotions they express about their situation

- Use descriptive emotional terms

**ADHERENCE ISSUES** - Identify medication adherence factors with supporting text

- mentions_discontinuation: Do they mention stopping statins completely?

- mentions_dose_changes: Do they mention changing their prescribed dose?

- mentions_compliance_issues: Do they mention difficulty following their prescription?

CRITICAL INSTRUCTIONS:

- Extract what is EXPLICITLY mentioned - don't infer beyond the text

- Use descriptive, flexible language rather than rigid categories

- Focus on clinically relevant information that would help healthcare providers

- Maintain objectivity while capturing the person's perspective

- Be precise but comprehensive in your extraction

- For all boolean fields, provide supporting text evidence when true

Return ONLY this JSON structure (no additional text):

{

"primary_themes": ["descriptive theme 1", "descriptive theme 2"],

"statin_mentioned": ["specific statin names if mentioned"],

"experience_type": "personal_experience",

"sentiment_detailed": {

"overall": "negative",

"towards_statins": "negative",

"towards_doctors": "neutral",

"towards_pharmaceutical_industry": "not_mentioned"

},

"specific_concerns": ["specific concern 1", "specific concern 2"],

"side_effects_mentioned": ["specific adverse effect 1", "specific adverse effect 2"],

"decision_factors": ["factor 1", "factor 2"],

"information_seeking": {

"asking_for_advice": {

"present": true,

"evidence": "exact quote or summary showing they're asking for advice"

},

"sharing_experience": {

"present": true,

"evidence": "exact quote or summary showing they're sharing experience"

},

"seeking_alternatives": {

"present": false,

"evidence": ""

},

"questioning_necessity": {

"present": false,

"evidence": ""

}

},

"clinical_relevance": {

"mentions_lab_values": {

"present": true,

"evidence": "specific lab values mentioned: LDL 180, etc."

},

"mentions_cardiovascular_events": {

"present": false,

"evidence": ""

},

"mentions_family_history": {

"present": true,

"evidence": "exact quote about family history"

},

"mentions_lifestyle_factors": {

"present": true,

"evidence": "specific lifestyle factors mentioned"

},

"actionable_insights": "medium"

},

"alternative_treatments": ["specific alternative 1"],

"emotional_indicators": ["emotion 1", "emotion 2"],

"adherence_issues": {

"mentions_discontinuation": {

"present": false,

"evidence": ""

},

"mentions_dose_changes": {

"present": false,

"evidence": ""

},

"mentions_compliance_issues": {

"present": true,

"evidence": "exact quote showing compliance issues"

}

}

}

```
