# Supplementary material for: Large Language Model–Based Analysis of Statin Therapy Discussions and Sentiment on Social Media: Cross-Sectional Observational Study
Source: J Med Internet Res. 2026 Apr 10;28:e85057. doi: 10.2196/85057 (PMC13068305; doi:10.2196/85057)
Supplement: Multimedia Appendix 3 [file jmir-v28-e85057-s003.docx]

Multimedia Appendix 3

**Expert Validation Protocol**

Reddit Statin Analysis: Expert Validation Framework

========================================================================

CONTENTS:

----------

1. Validation Overview

2. Hierarchical Thematic Framework

3. Extraction Domains and Definitions

4. Validation Protocol

5. Complete Example with Analysis

6. Validation Assessment Form

7. Scoring Guidelines

8. Documented Coding Challenges from Expert Validation

1. VALIDATION OVERVIEW

======================

PURPOSE:

This validation protocol ensures the accuracy and reliability of our GPT-4.1-based content analysis framework for Reddit statin discussions. Expert validators assess the performance of our automated extraction system across 12 analytical domains and hierarchical thematic categorization.

VALIDATION APPROACH:

• Stratified random sampling across key analytical domains

• Multi-domain assessment covering content and thematic analysis

• Evidence-based validation with original text quotes

• Hierarchical thematic mapping to manuscript framework

• 5-point Likert scale assessment (1=Poor, 5=Excellent)

• Qualitative feedback for improvement identification

SAMPLE COMPOSITION:

• High Clinical Relevance: 10 samples (20%)

• Adverse effects Mentioned: 15 samples (30%)

• Adherence Issues: 10 samples (20%)

• Negative Sentiment: 8 samples (16%)

• General Sample: 7 samples (14%)

• Total: 50 samples

2. HIERARCHICAL THEMATIC FRAMEWORK

====================================

Our analysis identified 14,007 total thematic mentions across 5,328 Reddit posts and comments, organized into a hierarchical framework:

2.1 TOPIC CATEGORIES (n=14,007 thematic mentions):

--------------------------------------------------

1. Medication Effectiveness and Efficacy (15.0%, n=2,104)

• Statin efficacy in cholesterol reduction

• Treatment outcomes and therapeutic benefits

• Comparative effectiveness discussions

2. Alternative Treatments and Lifestyle (14.9%, n=2,081)

• Dietary modifications (keto, plant-based, etc.)

• Exercise and lifestyle interventions

• Natural supplements and non-pharmaceutical approaches

3. Adverse effects and Adverse Reactions (14.2%, n=1,989)

• Muscle-related symptoms (myalgia, weakness)

• Cognitive effects (brain fog, memory issues)

• Other adverse reactions and tolerability concerns

4. Doctor-Patient Communication (8.2%, n=1,149)

• Healthcare provider interactions and recommendations

• Medical consultation experiences

• Communication barriers and satisfaction

5. Treatment Decisions and Hesitancy (5.6%, n=779)

• Statin initiation decisions

• Treatment hesitancy and reluctance

• Decision-making processes and uncertainty

6. Cardiovascular Events and Conditions (4.7%, n=659)

• Heart attacks, strokes, and cardiac events

• ASCVD risk and cardiovascular conditions

• Clinical presentations and outcomes

7. Laboratory Values and Monitoring (3.1%, n=428)

• Cholesterol levels (LDL, HDL, triglycerides)

• Lipid panel interpretations

• Monitoring protocols and frequency

8. Treatment Adherence and Compliance (2.7%, n=377)

• Medication adherence challenges

• Discontinuation and dose modifications

• Compliance strategies and barriers

9. Family History and Genetics (2.5%, n=347)

• Familial hypercholesterolemia

• Genetic risk factors and hereditary conditions

• Family cardiovascular history

10. Cost and Access Issues (1.9%, n=266)

• Medication costs and insurance coverage

• Access barriers and affordability concerns

• Healthcare accessibility issues

2.2 OVERARCHING THEMATIC GROUPS:

---------------------------------

1. Treatment Effectiveness and Management (23.6%, n=3,311)

• Combines: Medication Effectiveness, Laboratory Values, Treatment Decisions

2. Safety and Tolerability Concerns (16.9%, n=2,366)

• Combines: Adverse effects, Treatment Adherence

3. Alternative Approaches and Lifestyle (14.9%, n=2,081)

• Includes: Alternative Treatments and Lifestyle modifications

4. Healthcare System and Communication (10.1%, n=1,415)

• Combines: Doctor-Patient Communication, Cost and Access

5. Clinical Risk Factors (7.2%, n=1,006)

• Combines: Cardiovascular Events, Family History and Genetics

3. EXTRACTION DOMAINS AND DEFINITIONS

=====================================

3.1 PRIMARY THEMES

Identify 1–3 main discussion topics per post/comment. Use descriptive phrases drawn from the text, not a fixed list.

3.2 STATIN MEDICATIONS

Specific statin drugs mentioned (atorvastatin, simvastatin, rosuvastatin, etc.).

Includes brand names and generic formulations.

3.3 EXPERIENCE TYPE

Classification of user's relationship to content:

- personal_experience: Sharing one’s own statin journey or symptoms.
- information_request: Asking questions or seeking factual information.
- medical_advice: Providing recommendations or guidance to others.
- general_discussion: Broad commentary or theoretical discussion not seeking advice.

3.4 SENTIMENT ANALYSIS (MULTI-TARGET)

Emotional tone assessment across four targets:

- Overall sentiment (positive, neutral, negative, or mixed)
- Sentiment toward statins
- Sentiment toward doctors/healthcare providers
- Sentiment toward pharmaceutical industry

3.5 SPECIFIC CONCERNS

Particular worries or issues raised about statin therapy.

3.6 ADVERSE EFFECTS

Identify and categorize any adverse reactions reported.

3.7 DECISION FACTORS

Elements influencing statin therapy decisions:

• Laboratory results (cholesterol levels)

• Physician recommendations

• Adverse effect experiences

• Family history and genetic factors

• Cost and insurance considerations

3.8 INFORMATION-SEEKING BEHAVIORS

Evidence-based assessment of user information needs:

• Asking for advice

• Sharing experience to help others

• Seeking alternatives to statins

• Questioning necessity of treatment

3.9 CLINICAL RELEVANCE

Assessment of clinical actionability and relevance:

• Laboratory values mentioned

• Cardiovascular events discussed

• Family history relevance

• Lifestyle factors considered

• Overall actionable insight level (low, moderate, high)

3.10 ADHERENCE ISSUES

Treatment compliance challenges:

• Discontinuation mentions

• Dose changes or modifications

• General compliance issues

3.11 ALTERNATIVE TREATMENTS

Non-statin approaches discussed:

• Dietary interventions

• Exercise programs

• Natural supplements

• Other pharmaceutical alternatives

3.12 EMOTIONAL INDICATORS

Emotional states and psychological aspects:

• Anxiety, worry, fear

• Frustration, anger

• Hope, optimism

• Uncertainty, confusion

4. VALIDATION PROTOCOL

======================

4.1 PREPARATION PHASE

• Review hierarchical thematic framework

• Familiarize with extraction domain definitions

• Understand evidence-based assessment approach

• Review scoring guidelines (Section 7)

4.2 VALIDATION PROCESS

For each validation sample:

1. Read original Reddit text carefully and completely

2. Review LLM analysis output for all 12 domains

3. Assess thematic categorization accuracy

4. Verify evidence text quotes and appropriateness

5. Check hierarchical framework mapping

6. Rate accuracy using 5-point scale

7. Provide detailed qualitative feedback

8. Identify novel themes not captured in framework

4.3 KEY ASSESSMENT AREAS

• Completeness: Did LLM capture all relevant themes/content?

• Accuracy: Are classifications correct and appropriate?

• Evidence Quality: Are quotes relevant and supportive?

• Framework Mapping: Do themes map correctly to categories?

• Novel Content: Are there uncategorized themes present?

5. COMPLETE VALIDATION EXAMPLE

===============================

EXAMPLE SAMPLE ANALYSIS:

-------------------------

Document Number: 2286

Subreddit: r/keto

Document Type: comment

Date: 2025-04-16T22:34:05

Score: 2

ORIGINAL TEXT:

---------------

i am one of those people whose LDL skyrockets on keto. my triglycerides are good, HDL is good. I exercise frequently and my LDL on keto seems to fluctuate according to how hard i exercise (LDL increases the day after exercise). to decide about statins, i am trying to gather more info. LDL is one of several factors to consider when assessing your overall CVD risk. i ve had NMR lipid profile done to understand my LDL better (i have the pattern a , low CVD risk), a CRP test (low inflammation, expected on keto, low CVD risk), and a lipoprotein (a) test (moderate genetic risk for CVD) so all in all generally favorable for CVD risk. so for the time being, i am not taking statins and i am staying keto. i plan on getting a CAC score at some point this year, to use as a baseline. i can check again in a few years to see if hard plaque is building up. i m not sure if my LDL is a cause for concern by itself, when other risk factors look generally good. A poor CAC score might convince me to take statins however. LDL 298 pattern A HDL 77 TRI 55 CRP .4 LP(a) 115

LLM ANALYSIS OUTPUT:

====================

1. PRIMARY THEMES + HIERARCHICAL MAPPING:

LLM-Extracted Themes:

• personalized cardiovascular risk assessment

• decision-making about statin therapy

• impact of ketogenic diet and exercise on cholesterol

Mapped to Framework:

Topic Categories:

✓ Alternative Treatments and Lifestyle

✓ Treatment Decisions and Hesitancy

✓ Cardiovascular Events and Conditions

Overarching Groups:

✓ Treatment Effectiveness and Management

✓ Alternative Approaches and Lifestyle

✓ Clinical Risk Factors

2. STATIN MEDICATIONS:

None specifically mentioned

3. EXPERIENCE TYPE:

personal_experience

4. SENTIMENT ANALYSIS:

Overall: neutral

Toward Statins: neutral

Toward Doctors: not_mentioned

Toward Pharma: not_mentioned

5. SPECIFIC CONCERNS:

• LDL is one of several factors to consider when assessing your overall CVD risk

• i m not sure if my LDL is a cause for concern by itself, when other risk factors look generally good

6. ADVERSE EFFECTS:

None mentioned

7. DECISION FACTORS:

• lab results

• genetic risk (lipoprotein a)

• inflammation markers (CRP)

• planned CAC score

• exercise habits

• diet (keto)

8. INFORMATION-SEEKING (WITH EVIDENCE):

• Asking For Advice: ✓ YES

Evidence: "to decide about statins, i am trying to gather more info"

• Sharing Experience: ✓ YES

Evidence: "i am one of those people whose LDL skyrockets on keto... so for the time being, i am not taking statins and i am staying keto"

• Seeking Alternatives: ✗ NO

• Questioning Necessity: ✓ YES

Evidence: "i m not sure if my LDL is a cause for concern by itself, when other risk factors look generally good"

9. CLINICAL RELEVANCE (WITH EVIDENCE):

• Mentions Lab Values: ✓ YES

Evidence: "LDL 298 pattern A HDL 77 TRI 55 CRP .4 LP(a) 115"

• Mentions Cardiovascular Events: ✗ NO

• Mentions Family History: ✓ YES

Evidence: "lipoprotein (a) test (moderate genetic risk for CVD)"

• Mentions Lifestyle Factors: ✓ YES

Evidence: "i exercise frequently... i am staying keto"

• Actionable Insights: high

10. ADHERENCE ISSUES (WITH EVIDENCE):

• Mentions Discontinuation: ✗ NO

• Mentions Dose Changes: ✗ NO

• Mentions Compliance Issues: ✓ YES

Evidence: "so for the time being, i am not taking statins and i am staying keto"

11. ALTERNATIVE TREATMENTS:

• keto diet

• exercise

12. EMOTIONAL INDICATORS:

• uncertainty

• cautiousness

6. VALIDATION ASSESSMENT FORM

==============================

6.1 QUANTITATIVE RATINGS (1-5 Scale)

-------------------------------------

THEMATIC ANALYSIS:

1. Primary Themes Extraction Accuracy: ____

2. Topic Category Mapping Accuracy: ____

3. Overarching Group Mapping Accuracy: ____

4. Novel Theme Identification: ____

CONTENT ANALYSIS:

5. Statin Medications Identification: ____

6. Experience Type Accuracy: ____

7. Sentiment Analysis Accuracy: ____

8. Specific Concerns Identification: ____

9. Adverse effects Identification: ____

10. Decision Factors Identification: ____

11. Alternative Treatments Identification: ____

12. Emotional Indicators Identification: ____

BEHAVIORAL ANALYSIS:

13. Information-Seeking Behavior Detection: ____

14. Clinical Relevance Assessment: ____

15. Adherence Issues Detection: ____

EVIDENCE QUALITY:

16. Text Evidence Appropriateness: ____

17. Quote Accuracy: ____

18. Classification-Evidence Alignment: ____

OVERALL PERFORMANCE:

19. Overall LLM Performance: ____

6.2 QUALITATIVE FEEDBACK

-------------------------

A. Missed Themes/Categories:

______________________________________________________________________

______________________________________________________________________

B. Incorrect Classifications:

______________________________________________________________________

______________________________________________________________________

C. Evidence Quality Issues:

______________________________________________________________________

______________________________________________________________________

D. Novel Themes Identified (not in framework):

______________________________________________________________________

______________________________________________________________________

E. Framework Mapping Problems:

______________________________________________________________________

______________________________________________________________________

F. Overall Comments and Suggestions:

______________________________________________________________________

______________________________________________________________________

______________________________________________________________________

7. SCORING GUIDELINES

=====================

5-POINT LIKERT SCALE:

----------------------

5 = EXCELLENT: Perfect or near-perfect accuracy

• All relevant content captured accurately

• Appropriate classifications and mappings

• High-quality evidence text

4 = GOOD: High accuracy with minor issues

• Most content captured correctly

• Classifications mostly appropriate

• Minor evidence or mapping issues

3 = FAIR: Moderate accuracy with notable issues

• Some important content missed or misclassified

• Several classification or mapping errors

• Evidence quality concerns

2 = POOR: Low accuracy with significant problems

• Major content missed or misclassified

• Frequent classification errors

• Poor evidence selection

1 = VERY POOR: Unacceptable accuracy

• Most content missed or grossly misclassified

• Systematic classification failures

• Inappropriate or missing evidence

8. DOCUMENTED CODING CHALLENGES FROM EXPERT VALIDATION

=====================================================

During expert validation of the 50-discussion stratified sample, reviewers identified the following recurrent coding challenges. A representative case illustrating both challenges is provided below (see also Multimedia Appendix 4, Example 2a).

**Challenge 1: Genetic Biomarkers Misclassified as Family History**
The LLM occasionally conflated genetically influenced biomarkers with reported familial cardiovascular events. In a representative example, a user reported undergoing lipoprotein(a) testing and described “moderate genetic risk for CVD” based on laboratory results (Lp[a]=115). The model coded this as “Mentions Family History: YES.” However, Lp(a) is a laboratory-derived biomarker with high heritability and does not constitute family history unless accompanied by an explicit report of cardiovascular disease in relatives. The correct classification is “Mentions Family History: NO,” with the Lp(a) value captured under laboratory measures and genetic risk indicators.

**Challenge 2: Cardiovascular Testing Content Not Captured by Event-Oriented Coding**
References to cardiovascular testing and planned risk stratification were sometimes not captured. In the same example, the user described obtaining a coronary artery calcium (CAC) score “as a baseline,” monitoring for “hard plaque building up,” and noted that an unfavorable CAC result might influence statin initiation. The model coded “Mentions Cardiovascular Events: NO.” While this is correct because CAC testing is not an event, the post contains clinically relevant cardiovascular assessment content that should be represented (eg, under a “Cardiovascular testing/assessment” category). Omitting such content may underestimate the clinical relevance of discussions centered on risk evaluation and decision-making.
