# Supplementary material for: Large Language Model–Based Analysis of Statin Therapy Discussions and Sentiment on Social Media: Cross-Sectional Observational Study
Source: J Med Internet Res. 2026 Apr 10;28:e85057. doi: 10.2196/85057 (PMC13068305; doi:10.2196/85057)
Supplement: Multimedia Appendix 4 [file jmir-v28-e85057-s004.docx]

Exemplar Posts with LLM-Derived Theme and Sentiment Classifications

Theme 1: Adverse effects and Adverse Reactions

Example 1a: "My doctor started me on [a statin] and last night I took my first pill. This morning I woke up with a pain in the chest and gas. I'm guessing heartburn? I took the pill around 7pm since I was scared of adverse effects, I didn't eat and only had ginger water to drink. [...] I'm just wondering if there's something I can do to fix this? Maybe take it at a different time? [...] just hate this feeling"

LLM Prediction: Themes identified as adverse effects and medication timing. Overall sentiment negative. Sentiment toward statins negative. Adverse effects identified include chest pain, heartburn, and gas. Information seeking flagged as asking for advice and sharing experience. Adherence flagged for compliance issues.

Example 1b: "Anyone have leg issues with [a statin]? Lately I feel like my legs (calves and ankles) have been through major workouts. Calves feel super tight and ankle areas don't feel the greatest either. Just wondering if anyone has been through this and what did you do to fix it?"

LLM Prediction: Themes identified as experiencing muscle-related adverse effects from statin and seeking advice from others with similar experiences. Overall sentiment negative. Sentiment toward statins negative. Adverse effects identified include muscle tightness and leg discomfort. Information seeking flagged as asking for advice. Experience type classified as personal experience.

Theme 2: Decision-Making -- Laboratory Results

Example 2a: "I am one of those people whose LDL skyrockets on keto. [...] To decide about statins, I am trying to gather more info. LDL is one of several factors to consider when assessing your overall CVD risk. I've had NMR lipid profile done to understand my LDL better (I have the pattern A, low CVD risk), a CRP test (low inflammation [...] low CVD risk), and a lipoprotein(a) test (moderate genetic risk for CVD) so all in all generally favorable for CVD risk. So for the time being, I am not taking statins [...] I plan on getting a CAC score [...] as a baseline. [...] A poor CAC score might convince me to take statins however. LDL [near 300] pattern A, HDL [high 70s], TRI [mid 50s], CRP [<1], LP(a) [elevated]"

LLM Prediction: Themes identified as personalized cardiovascular risk assessment, decision-making about statin therapy, and impact of ketogenic diet on cholesterol. Overall sentiment neutral. Sentiment toward statins neutral. Decision factors include lab results, genetic risk (Lp(a)), inflammation markers (CRP), planned CAC score, exercise habits, and diet. Clinical relevance rated high. Experience type classified as personal experience.

Example 2b: "My results after [approximately 2 weeks] on [a statin] having lp(a) [elevated]. Total cholesterol: from [~130] to [~100]. [...] LDL: from [~65] to [~40]. All values are in mg/dl. I am going to discuss about adding ezetimibe with reducing [statin dose]. All liver and kidney tests came back normal. [...] I am still targeting LDL-C around 30. Would [lower-dose statin] and 10mg ezetimibe [be] enough?"

LLM Prediction: Themes identified as cholesterol management and statin therapy adjustment. Overall sentiment neutral. Sentiment toward statins neutral. Decision factors include lab results and dose optimization. Clinical relevance flagged for lab values. Adherence flagged for dose changes. Information seeking flagged as asking for advice.

Theme 3: Decision-Making -- Physician Advice

Example 3a: "My doc seems hesitant to put me on statins, but it's been 3 years high -- thoughts? [...] My cholesterol has remained high for 3 years despite lifestyle changes, but doc keeps pushing out statins and I'm feeling worried. [...] I had a really complicated pregnancy [...] I developed preeclampsia [...] and gestational diabetes. [...] This last year I've worked my butt off. Reading everything, trying everything, tracking. Giving up so many foods. Working out loads. I only managed to get my total cholesterol down from [~6.1] to [~5.6 mmol/L] [...] I sincerely don't think I can change my lifestyle any further at this point. [...] I guess I am just wanting to hear from the folks who tried everything and still needed statins."

LLM Prediction: Themes identified as persistent high cholesterol and hesitancy about statin therapy. Overall sentiment negative. Decision factors include physician advice, lab results, family history, and lifestyle factors. Clinical relevance flagged for lab values, family history, and lifestyle factors. Information seeking flagged as asking for advice and sharing experience.

Example 3b: "My total [cholesterol] is [nearly 290]. LDL [over 200]. I'm [in my early 50s]. Dr said he'd recheck me in six [months]. Cut out cheese [...] fats etc. No advice on statins but your numbers are so much lower than mine and your doc wanted medicine. But mine is like... see ya in six [months]."

LLM Prediction: Themes identified as differences in physician approach to high cholesterol, lack of statin recommendation despite high cholesterol, and lifestyle modification advice. Overall sentiment negative. Sentiment toward statins neutral. Decision factors include doctor recommendation and lab results. Mapped categories include Alternative Treatments and Lifestyle and Doctor-Patient Communication.

Theme 4: Information Seeking

Example 4a: "I ([late 60s, male]) have been on [a statin] for about 10 years. [...] My cardiologist recently added [ezetimibe]. [...] No heart problems, etc. Passed all my tests including my stress test. But he still added [ezetimibe] which brought my cholesterol down dramatically. My GP questioned why my cardiologist added [it]. No one I know who takes cholesterol meds even heard of [ezetimibe]. Are any of you guys taking [it]? Alone or in combo of other meds?"

LLM Prediction: Themes identified as medication management and doctor-patient communication. Overall sentiment neutral. Information seeking flagged as asking for advice, sharing experience, and questioning necessity. Adherence flagged for dose changes. Experience type classified as personal experience.

Example 4b: "How many people are on statins? Just out of curiosity, I was wondering how many people with CKD are prescribed statins by their nephrologist. I've had CKD for [over a decade] and I've been taking [a statin] for all [that time]. However, I was already on this from my primary care doctor previously, so I am unsure if my nephrologist would prescribe it if I wasn't already taking it."

LLM Prediction: Themes identified as statin prescription in CKD and doctor prescribing practices. Overall sentiment neutral. Sentiment toward statins neutral. Information seeking flagged as asking for advice and sharing experience. Clinical relevance rated as low actionable insights. Experience type classified as personal experience.

Theme 5: Alternative Approaches and Lifestyle

Example 5a: "My cholesterol had been trending higher over the years [...] I decided to really make some diet changes and add a few supplements [...] I basically cut out anything above like 2-3g saturated fat in a serving. [...] Started taking red yeast rice extract along with CoQ10 daily. Red yeast rice contains monacolin K, a compound also found in some statin medications. [...] Started taking omega-3 fish oil [...] psyllium husk powder [...] I basically began following a more Mediterranean type diet. [...] Here are my [before vs. after] bloodwork changes: LDL: [~180] -> [~120] (mg/dL) [...] Triglycerides: [~100] -> [~35] (mg/dL) [...] Total Cholesterol: [~245] -> [~175] (mg/dL)"

LLM Prediction: Themes identified as dietary modification and supplement use. Overall sentiment positive. Clinical relevance flagged for lab values and lifestyle factors with high actionable insights. Information seeking flagged as sharing experience and seeking alternatives. Alternative treatments identified include red yeast rice, CoQ10, fish oil, psyllium, and Mediterranean diet.

Example 5b: "[I was on a] statin for high LDL. HDL was super low. On blood pressure meds, blood thinners, acid reflux meds. A1C was just a [tick] below pre-diabetic. [...] Had severe sleep apnea. Went on a [meat-based] diet [...] Lost [~100] lbs. HDL and triglycerides are great. I'm off blood pressure meds [...] A1C is now [normal]. Off blood thinners. Sleep apnea is gone. The ONLY marker that is not good is my LDL. It is high. And I am absolutely not worrying about it. The most recent science does not support the old paradigm that lower LDL is better. There is plenty of evidence that when all other metabolic markers are in the proper range, elevated LDL is actually a great thing for your mind and body."

LLM Prediction: Themes identified as dramatic health improvements through diet and weight loss, skepticism about the importance of lowering LDL cholesterol, and discontinuation of multiple medications after lifestyle changes. Overall sentiment positive. Sentiment toward statins negative. Decision factors include lab results and online research. Mapped categories include Medication Effectiveness and Efficacy and Alternative Treatments and Lifestyle.

Theme 6: Adherence-Related Issues

Example 6a: "Yeah, I haven't gone back to it. My lipid panel had my LDL at [~115] when I told my doc I couldn't do statins anymore. With diet and exercise, I'm at [under 90]. [Triglycerides] are optimal again. And I had a calcium [score] test [...] that came back 0."

LLM Prediction: Themes identified as cholesterol management and statin intolerance. Overall sentiment positive. Adherence flagged for discontinuation. Clinical relevance flagged for lab values and lifestyle factors. Information seeking flagged as sharing experience.

Example 6b: "Will going down from [higher dose] temporarily to [lower dose] make a big LDL difference? [...] I had been on [lower-dose statin] when my LDL was about [~195]. It was [~75] in one test and then [...] a couple months later was [~115]. My doctor decided to up my statin dose [...] I am going to be starting [a PCSK9 inhibitor] tomorrow and going back down to the [lower-dose statin] because I was getting adverse effects from increased dosage. Will being on the [higher dose] for a week or so and then going back down [...] make my numbers worse? I didn't know if it was something where once your body gets used to the [higher dose] if you go back down [...] it doesn't work as well."

LLM Prediction: Themes identified as concerns about LDL control with statin dose changes and managing statin adverse effects and medication adjustments. Overall sentiment mixed. Sentiment toward statins negative. Adverse effects flagged from increased dosage. Adherence flagged for dose changes. Decision factors include adverse effects, doctor recommendation, and lab results. Mapped categories include Adverse effects and Adverse Reactions, Treatment Adherence and Compliance, and Laboratory Values and Monitoring.
