# Supplementary material for: Large Language Model–Based Analysis of Statin Therapy Discussions and Sentiment on Social Media: Cross-Sectional Observational Study
Source: J Med Internet Res. 2026 Apr 10;28:e85057. doi: 10.2196/85057 (PMC13068305; doi:10.2196/85057)
Supplement: Multimedia Appendix 5 [file jmir-v28-e85057-s005.docx]

Multimedia Appendix 4

eTable 1. Frequency of Specific Adverse effects Mentioned in Reddit Discussions on Statin Therapy

| Symptom Category | Specific Adverse effect | No. of Mentions (n) | % of Adverse effect Discussions* | 95% Confidence Interval |
| --- | --- | --- | --- | --- |
| **Musculoskeletal** | Muscle Pain | 129 | 7.6% | 6.4% - 8.9% |
|  | Muscle Aches | 33 | 1.9% | 1.3% - 2.7% |
|  | Muscle Weakness | 23 | 1.4% | 0.9% - 2.1% |
| **General/Systemic** | Fatigue | 110 | 6.5% | 5.4% - 7.7% |
|  | Dizziness | 40 | 2.4% | 1.7% - 3.2% |
|  | Nausea | 33 | 1.9% | 1.3% - 2.7% |
|  | Headaches | 30 | 1.8% | 1.2% - 2.5% |
|  | Diarrhea | 28 | 1.6% | 1.1% - 2.3% |
| **Cognitive** | Cognitive Effects ("Brain Fog") | 61 | 3.6% | 2.8% - 4.6% |
| **Neuropsychiatric** | Depression | 23 | 1.4% | 0.9% - 2.1% |
|  | Anxiety | 23 | 1.4% | 0.9% - 2.1% |
|  | Insomnia | 19 | 1.1% | 0.7% - 1.7% |
